# Serious Games for Improving Genetic Literacy and Genetic Risk Awareness in the General Public: Protocol for a Randomized Controlled Trial

**DOI:** 10.2196/resprot.9288

**Published:** 2018-12-18

**Authors:** Serena Oliveri, Renato Mainetti, Alessandra Gorini, Ilaria Cutica, Giulia Candiani, Nunzio Alberto Borghese, Gabriella Pravettoni

**Affiliations:** 1 Department of Oncology and Hemato-Oncology Università degli Studi di Milano Milan Italy; 2 Applied Intelligent Systems Laboratory Department of Computer Science Università degli Studi di Milano Milan Italy; 3 Zadig Srl Milan Italy

**Keywords:** adventure games, cardiovascular risk, decision making, genetic literacy, genetic risk, heredity, knowledge transfer, mini-games, mutation, serious games

## Abstract

**Background:**

Genetic testing and genetic risk information are gaining importance in personalized medicine and disease prevention. However, progress in these fields does not reflect increased knowledge and awareness of genetic risk in the general public.

**Objective:**

Our aim is to develop and test the efficacy of a suite of serious games, developed for mobile and Web platforms, in order to increase knowledge of basic genetic concepts and promote awareness of genetic risk management among lay people.

**Methods:**

We developed a new ad-hoc game and modified an arcade game using mechanics suitable to explain genetic concepts. In addition, we developed an adventure game where players are immersed in virtual scenarios and manage genetic risk information to make health-related and interpersonal decisions and modulate their lifestyle. The pilot usability testing will be conducted with a convenience sample of 30 adults who will be categorized into 3 groups and assigned to one game each. Participants will be asked to report any positive or negative issues arising during the game. Subsequently, they will be asked to complete the Game Experience Questionnaire. Finally, a total of 60 teenagers and adults will be enrolled to assess knowledge transfer. Thirty participants will be assigned to the experimental group and asked to play the serious games, and 30 participants will be assigned to the control group and asked to read leaflets on the genetic concepts conveyed by the games. Participants of both groups will fill out a questionnaire before and after the intervention to assess their topic-specific knowledge of genetics. Furthermore, both groups will complete the self-efficacy questionnaire, which assesses the level of confidence in using genetic information.

**Results:**

We obtained evidence of game usability in 2017. The data will be submitted to a peer-reviewed journal and used to improve the game design. Knowledge-transfer testing will begin in 2018, and we expect to collect preliminary data on the learning outcomes of serious games by December 2018.

**Conclusions:**

It is important to educate the general public about the impact of genetics and genetic testing on disease prevention and the consequent decision-making implications. Without such knowledge, individuals are more likely to make uninformed decisions or handover all decisions regarding genetic testing to their doctors. Technological innovations such as serious games might become a valid instrument to support public education and empowerment.

**International Registered Report Identifier (IRRID):**

DERR1-10.2196/9288

## Introduction

### Background

Genetic tests identify changes in chromosomes, genes, or proteins that may be related to an increased probability of inheriting or developing a disorder or disease [1]. Over the last 20 years, genetic tests have gained importance in personalized medicine and disease prevention and are usually prescribed by physicians to healthy individuals who have a family history of disease or patients who may have a disease resulting from a genetic mutation [2]. Since 2002 [3], people have been able to autonomously purchase genetic tests from the internet or local private companies that sell direct-to-consumer (DTC) genetic tests, which allows them to determine their genetic predisposition to diseases. The increasing success of such services indicates that people wish to obtain health information on their own even when it is not strictly required by their physicians or warranted by their family predisposition to certain illnesses [4].

Proponents of the DTC genetic test services argue that allowing consumers to calculate their relative risk of developing certain diseases may result in increased patient awareness, improved compliance with health-screening practices, and a better ability to make healthy lifestyle choices [5,6]. Despite these expectations, available data on the effects of DTC genetic tests on consumers are not encouraging. People face difficulties in understanding genetic risk information and its implications for health: On receiving their genetic results, patients sometimes experience unnecessary anxiety and emotional distress or make decisions about their health based on incomplete information that often results in increased health care costs [5-7]. Also, the expected changes are rarely executed in the consumers’ lifestyle habits and usually restricted to the few weeks following the test [8-11]. Thus, the increased use of genetic tests does not reflect increased knowledge and awareness of genetic risk in the general public.

In the last few years, several attempts have been made to improve genetic knowledge among the public through different approaches such as the recent implementation of serious games like Touching Triton [12], Geniverse [13], and DNA Roulette [14]. Unfortunately, the current serious games on genetics are mostly intended for trainees in biology courses and medical practitioners (geneticists): They use technical language or focus on certain aspects of genetics such as the probabilistic nature of genomics and neglect the complexity of simplifying such information for the general public. The serious game approach is promising, as it represents a highly interactive medium that supplements traditional educational modalities. However, thus far, there are no available data on the effectiveness of existing serious games in increasing people’s knowledge about genetic concepts.

### Primary Aim

This study aimed to evaluate the effectiveness of serious games in terms of the learning outcomes in the fields of genetics and genetic risk. In particular, we aimed to assess the learning impact of a suite of mini-games on participants’ knowledge and understanding of basic genetic concepts and to determine the effect of an adventure game on participants’ skills and perceived self-efficacy with regard to genetic risk management.

### Secondary Aim

The secondary aim was to evaluate game-flow parameters for two mini-games and an adventure game.

## Methods

### Team and Target Population

Serious games were designed and developed by a multidisciplinary team of psycho-oncologists, computer scientists, and a science journalist. Through constructive discussion among different professionals in the field of genetics, accuracy and consistency of genetic contents such as a detailed description of heredity mechanisms were ensured. Genetic concepts were explained in a simple way and presented as interesting facts for laypeople, as described previously [15,16]. The choice of thematic concepts and game scenarios was based on the important cornerstones of genetics.

The target population comprised people interested in genetics, high-school students, and people who require or need to decide whether to undergo genetic counseling or a genetic test. The developed serious games are suitable for people aged 16-65 years, during which primary prevention has a high impact on the health status. Therefore, we recruited participants aged 16-65 years to include teenagers who start to learn more complex genetic principles (such as DNA structure, cell duplication, mutations, and gene interaction) during their high school years and older adults who are interested in playing games or still accustomed to playing games [17]. The research protocol was approved by the Institutional Review Board of the University of Milan and the Centre for Research Ethics & Bioethics at Uppsala University (leader of Mind the Risk project). The study was conducted according to the Helsinki Declaration.

### Game Design and Learning and Educational Aspects of Serious Games

The new generations are raised in a digital world and have a natural attitude toward the digital language of computers, video games, and the internet. Young people, in particular, spend a significant amount of time playing computer games through which, they usually experience a high level of motivation and engagement. To them, traditional learning is an incredibly boring, effort-intensive, complex task [18].

To improve lay people’s attention, motivation, and engagement in genetic concepts, challenging activities and clear educational goals were embedded in our games to guarantee pleasure and “flow” (a situation of complete absorption or engagement [19-21]), which are relevant dimensions for the efficacy of serious games.

Learning can be viewed as both information acquisition and knowledge construction (ie, the ability to use new knowledge). An individual’s ability to act appropriately in a given situation depends on his or her knowledge and knowledge-transfer ability in a particular situation. In our study, the serious games aimed to maximize genetic learning by presenting individuals with genetic information and having them apply this information to successfully proceed with the game.

**Figure 1:**
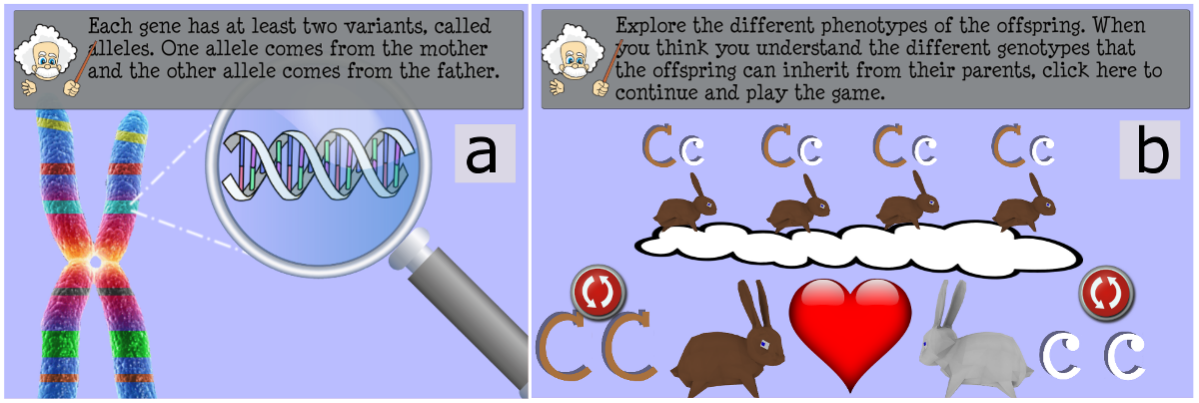
(a) An overview of basic genetic concepts (gene, alleles, genotype, and phenotype) is provided to the player before the game session. (b) The player can practice with Mendel's laws.

Several components of our games stimulate the learning process. First, genetic concepts are introduced and explained in a conversational manner by a virtual narrator (Figure 1) during an initial interactive tutorial. The virtual narrator—SCI (Scientist)—accompanies the player through the tutorial, during which, the characters, elements, and main rules of the game are introduced and specific genetic mechanisms are discussed. Thereafter, during the game sessions, information, hints, and feedback are provided to prevent players from getting stuck during the game (due to the lack of understanding) or to fix some concepts explained in the tutorial. Immediately after listening and interacting with the SCI, the player has to overcome challenges, wherein he or she applies the basic concepts of genetics. Thus, the player has the opportunity to practice genetic concepts in the application to win the game.

When creating the game, we balanced the complexity of genetic concepts, the game challenge, and the required cognitive load by introducing different levels of difficulty, thereby alternating moments of challenge and reflection and providing hints through the SCI. This approach provided the players sufficient time to reflect and revisit the rules of the genetic mechanisms explained in the tutorial.

To successfully proceed with the adventure game and maintain the principal character in good health (see Game Description), players need to apply their learnings from the game session to each specific situation in the narration. Another important aspect of the serious game design was the provision of clear immediate feedback (audio and video) that reflected user performance. The player interacts with the game on the basis of the new genetic mechanisms he or she learned during the tutorial (testing) and determines the result of this interaction through immediate feedback (revision). The feedback guarantees the ability to understand the results of the action taken during the play session.

To create the serious game, we followed Piaget’s Theory of Cognitive Development, with the principles of assimilation (the player fits “new information” about genetics into existing slots or categories he or she had before playing the game), accommodation (the player accommodates “new information” about genetics that does not fit into an existing slot or category), and cognitive disequilibrium (presence of contradictory beliefs) to support the learning process [22]. The challenges create a cognitive disequilibrium (a situation where new information is not immediately interpreted on the basis of existing categories), without exceeding the capacity of the player to succeed. The player tries to find a new equilibrium by modifying his or her cognitive patterns and incorporating the newly acquired knowledge.

### Game Description

Two mini-games and an adventure game were developed for this study.

For the mini-games, we used two different approaches. The first approach was the creation of ad-hoc serious games: We implemented a two-dimensional jump-and-run game to convey Mendel’s laws and incorporated genetic concepts into the game mechanics. The second approach was a modification of the mechanics of the arcade game Tetris to create a learning version of an existing game in order to represent genetic mutations. This approach may be familiar and appeal to most users, owing to the popularity of this arcade game [19,20].

The mini-games were designed exclusively to transmit basic genetic principles and improve general public literacy without revealing the genetic risk. These games are based on a simplified representation of mutations and Mendel’s laws. For example, in the serious game Mutan-Tetris, the game field represents the events that could occur in a cell’s life and their eventual consequences on the organism. In the serious game Heredi-Rabbit, the game challenges are established by the possible allelic combination following hereditary transmission.

For the adventure game, a narrative based on a dramatic curve was employed. The narrative can increase pleasure and provide background and motivation for the player to involve himself or herself in the game [21].

The adventure game was conceived to spread awareness about genetic risk. The player manages the genetic risk by identifying with an avatar and modifying his or her behavior according to his or her genetic predisposition.

All the developed games are currently available in Italian and English languages. They were developed mainly for mobile and Web platforms and will be available to the public on the official website of the Mind the Risk project (2019) during the last year of the project [23].

#### Heredi-Rabbit

The aim of this game is to explain concepts of heredity (the process through which genetic traits are passed from parents to their offspring), with dominant and recessive genetic variants (phenotype expression if the gene variant is present in at least one copy versus gene variant expression if the gene variant is present in both chromosomes; Mendel’s laws).

To help people with no prior knowledge in the field of genetics, a short tutorial is available before the game session. In this tutorial, several basic concepts of cells, chromosomes, genes, phenotype, and dominant and recessive traits are explained by the virtual narrator using text and simple animated illustrations. A brief introduction to the fundamental aspects of genetics (Figure 1a) and an optional practice exercise for Mendel’s law (Figure 1b) explains the core of the game to the player.

**Figure 2:**
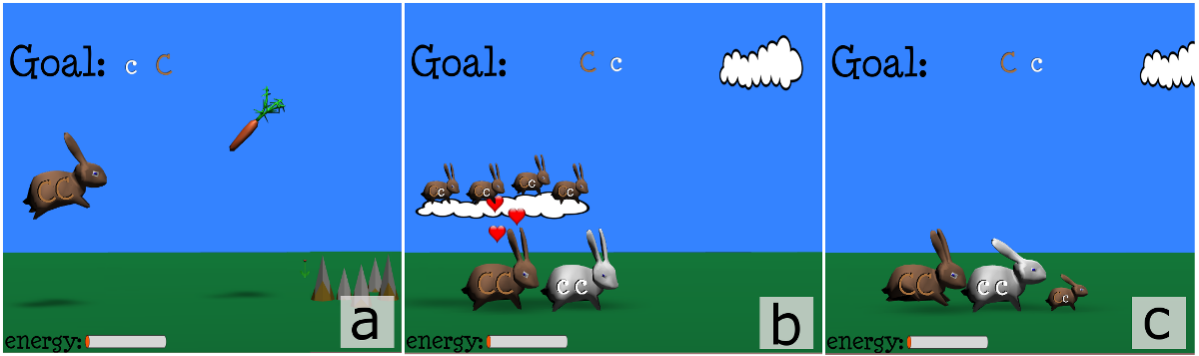
(a) The player controls the rabbit with the aim to grab carrots to gain energy and avoid traps; (b) The player can choose to mate the running rabbit with other incoming rabbits based on their phenotypes, giving birth to an offspring; (c) The newborn rabbit inherits its genetic make-up according to Mendel’s laws. When the targeted genetic make-up is achieved (goal), the game ends.

In this two-dimensional jump-and-run game, the player has to make a rabbit mate with other rabbits in order to birth an offspring with a specific genetic makeup (final goal of the game session). The rabbit hops around, grabs carrots to gain energy, avoids traps, and meets rabbits of the opposite sex (Figure 2a). The player can choose to mate the rabbit with the incoming rabbit based on their phenotypes, giving birth to an offspring (Figure 2b). In this phase of the game, the time allotted to the player to make a choice is limited (imposed pace), which maintains a high level of fun, engagement, and attention, since the player has to remember and apply the previously learned concepts quickly. As soon as the player makes a decision, the game is paused, the possible allelic combination and phenotype of the offspring are shown, and the player has time to reflect on Mendel’s laws (self-paced). After a brief pause, the game resumes, and a new rabbit is randomly selected from the offspring. The new-born rabbit that inherits the genetic makeup as per Mendel’s laws becomes the new runner rabbit (Figure 2c). The speed of the running rabbits can be regulated to optimize the time required to determine whether the incoming rabbit is appropriate for that game session goal. The game ends when the newborn running rabbit achieves the genotype goal specified at the beginning of every session.

#### Mutan-Tetris

The goal of this game is to explain the following aspects of mutations: definition of a mutation (an alteration of the nucleotide sequence in the DNA), factors that contribute to mutations (errors, mutagens, or environmental causes), fixable and unfixable mutations, hereditary mutations, and the increase in mutation rate with age.

**Figure 3:**
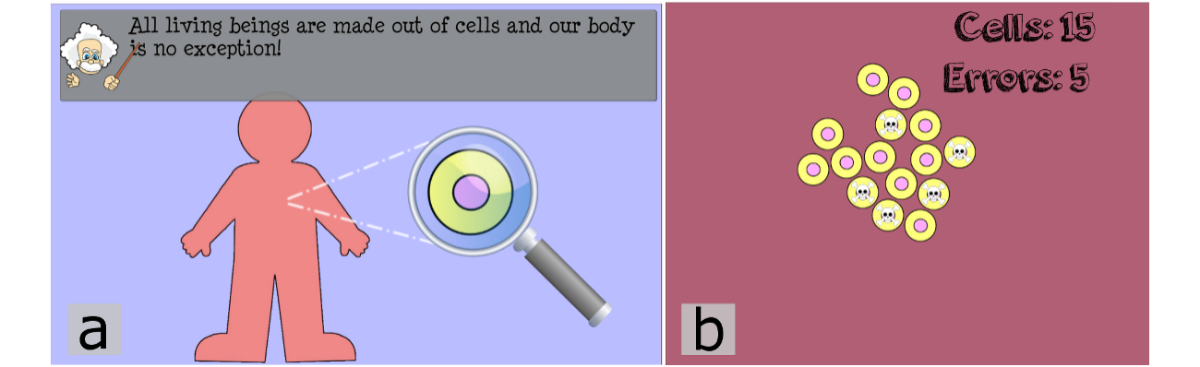
(a) The virtual narrator provides an introduction to the fundamental aspects of cell duplication and duplication errors using simple animated illustrations. (b) An interactive portion encourages the player to duplicate a predefined number of cells without duplicating cells containing errors.

In a simple introduction, the fundamental aspects of cell duplication (Figure 3a) and duplication errors (Figure 3b) are explained by the virtual narrator using text and simple animated and interactive illustrations.

**Figure 4:**
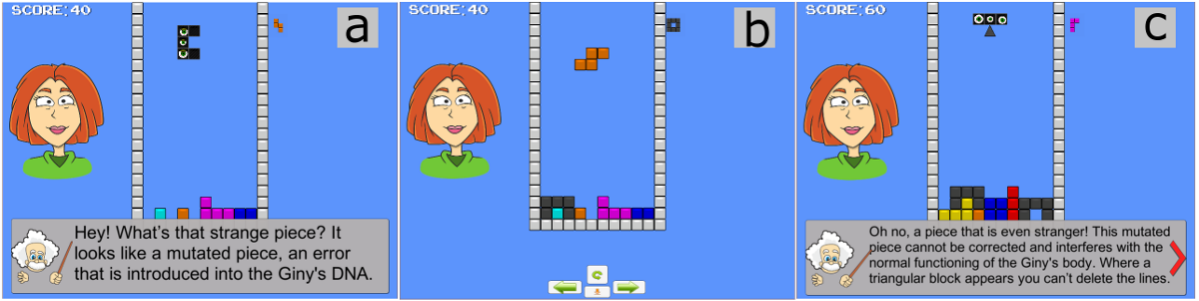
Mutant-Tetris. Classical Tetris is modified using several new mutated bricks. The bricks represent the genetic material, and elimination of a line represents correct DNA coding. (a, b) The two fixable mutated bricks increase the difficulty in deleting lines. (c) The mutated brick has a geometry that does not allow line deletion.

In the tutorial, the player learns that some mutations are fundamental because they provide genetic variability, which is the basis for evolutionary changes over time. However, the game focuses on harmful mutations, both somatic (any cell of the organism except reproductive cells, not usually transmitted to descendants) and germline (can be passed to the offspring through reproductive cells), that may adversely affect the function of a cell (mutations occurring in coding DNA). We used a famous arcade game, Tetris, and modified its mechanics by adding bricks with new shapes (Figure 4). In the classical Tetris version, 7 different bricks exist, each with its own shape and color. Bricks fall from the top of the screen and the player has to rotate and shift them in the best way during the fall to form full lines at the bottom and prevent leaving empty spaces. The amount of time allotted to identify the best position and orientation of each brick is limited (imposed pace) in order to maintain the challenge and engagement. Every time a line fills up, it is erased, and the player gains points. In our version, the Tetris environment metaphorically represents a cell environment, bricks represent genetic material, and deleted rows represent correct DNA reading. Anyone who has played Tetris is familiar with the shape of the 7 bricks. In this game, we have introduced 3 new bricks that represent mutations, with different shapes, a blinking-eye icon in the middle, and dark colors. These bricks aim to warn the player of unexpected and unusual events. When such bricks appear, the game is paused (self-paced) and the narrator explains the origin of the “mutation” in detail. When the player has understood the brick, the game is resumed. Two of the new bricks are more difficult to use for deleting lines, but the player can still fit them in with other bricks and then delete rows: These bricks represent mutations that can be auto-fixed by our cells (Figure 4a and b). The third brick has a geometry that does not fit in the existing lines without leaving any space: The line cannot be completely deleted. Every time one of these bricks is introduced, one row of the Mutan-Tetris becomes indestructible, reinforcing the message that not all mutations can be fixed by our cells (Figure 4c). The game has been parameterized with several levels of difficulty, which modify the probability of appearance of the new “mutated” bricks and allow the player to explore all the serious game contents. If the player is unable to achieve the minimum score required to trigger the appearance of the mutated pieces, the player is informed that the level chosen is too difficult and is recommended to retry the game at an easier level, following which the game restarts. The game ends when no more lines can be filled or deleted and there is no space for new bricks in the playing field. The entire playing field filled with unfixed or damaging mutations represents an incoming condition that could affect “cell health.”

#### Adventure Game

**Figure 5:**
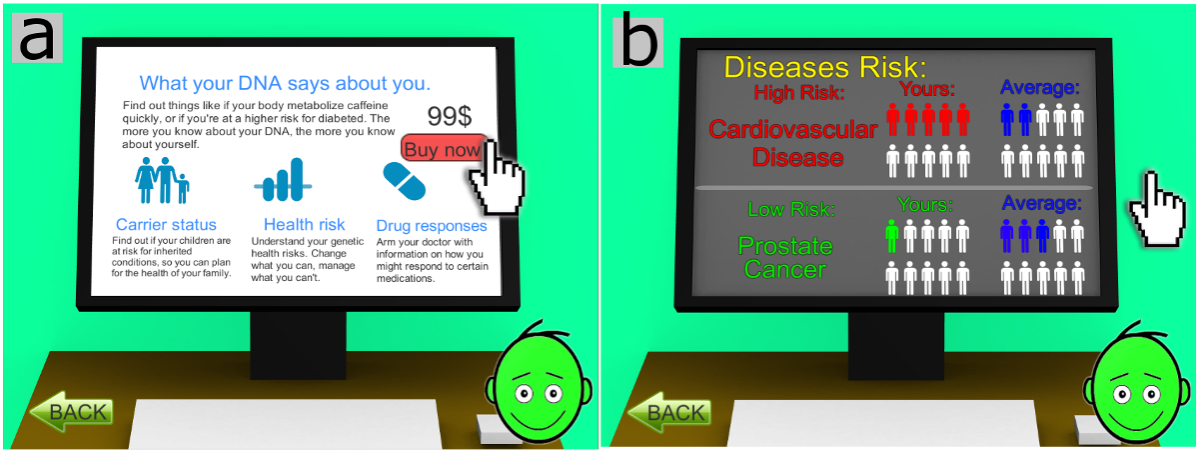
(a) Eugene is going to buy a direct-to-consumer genetic test online. (b) He receives the test results and discovers he is at high risk for cardiovascular disease.

Gene Adventure, the World of Tomorrow, is an adventure game, wherein the player embodies a young adult named Eugene who lives his everyday life after undergoing DTC genetic testing (Figure 5a). The game takes place in a small city with several facilities (markets, restaurants, pubs, parks, etc). The dramatic curve of Eugene’s story guides the player through several events and answers the “why” of the game, stimulating involvement and motivation. During the game, Eugene learns about the presence of some gene variants and his risk of developing cardiovascular diseases (Figure 5b).

**Figure 6:**
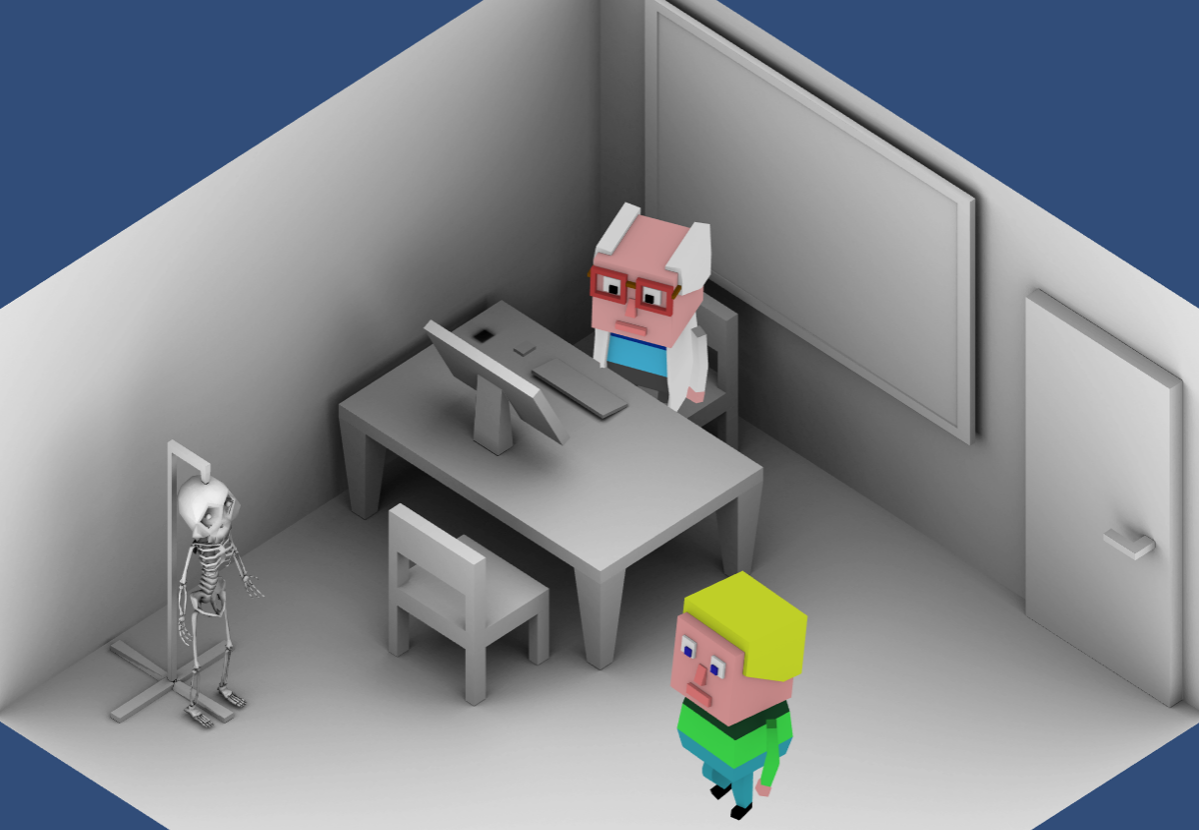
The player can decide whether Eugene has to talk to his doctor about his genetic risk for cardiovascular disease.

Unfortunately, he cannot manage this information. He is given several chances to obtain clarifications about the implications of the genetic result and its consequences on his health. The player must help Eugene find the best way to manage his cardiovascular risk by accomplishing several subtasks, making health-related decisions (Figure 6), modulating his lifestyle, and interacting with other characters.

**Figure 7:**
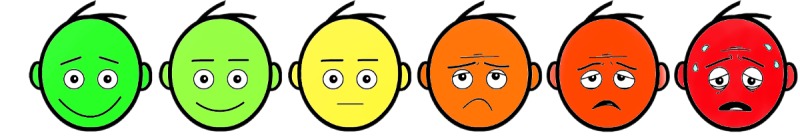
The colors and expressions of the “smile icon” represent Eugene’s health status.

The player is given three options for every choice throughout the game session, which are incorporated into the avatar’s behaviors, and each option is assigned a score as follows: –1 for the unhealthy choice, 0 for the neutral choice, and +1 for the healthy choice. The chosen option is simultaneously translated into clear feedback as a modification of the color and expression of a “smile icon,” which represent Eugene’s health state and quality of life (Figure 7).

The three alternatives for each choice are formulated to reflect either recurring behaviors (habits) or occasional behaviors (occurrence) to show that some behaviors could be problematic if repeated over time, and other behaviors are problematic even they are occasional.

### In-Game Assessment of Knowledge Transfer and Learning Progress

**Figure 8:**
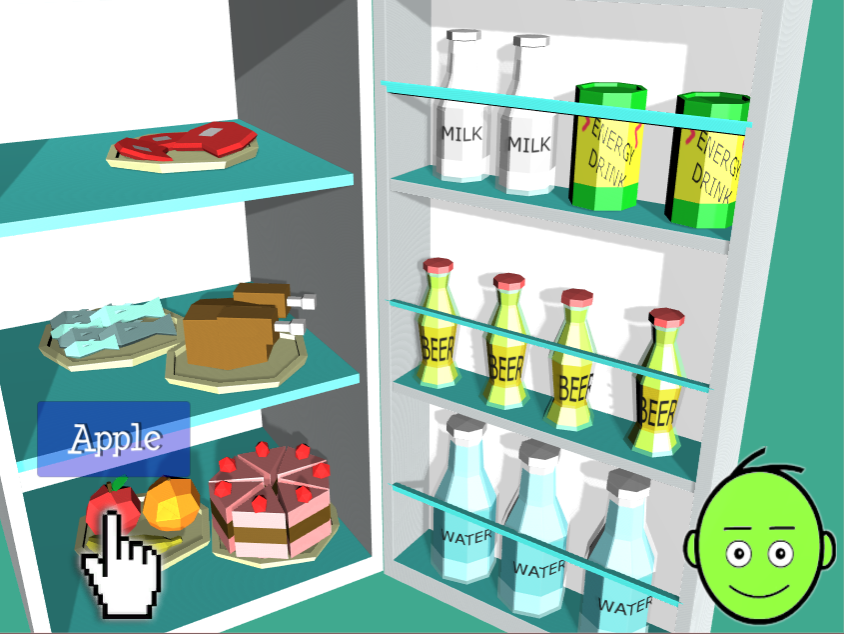
During the game, the player carefully chooses Eugene’s diet to maintain correct nutrition habits.

**Figure 9:**
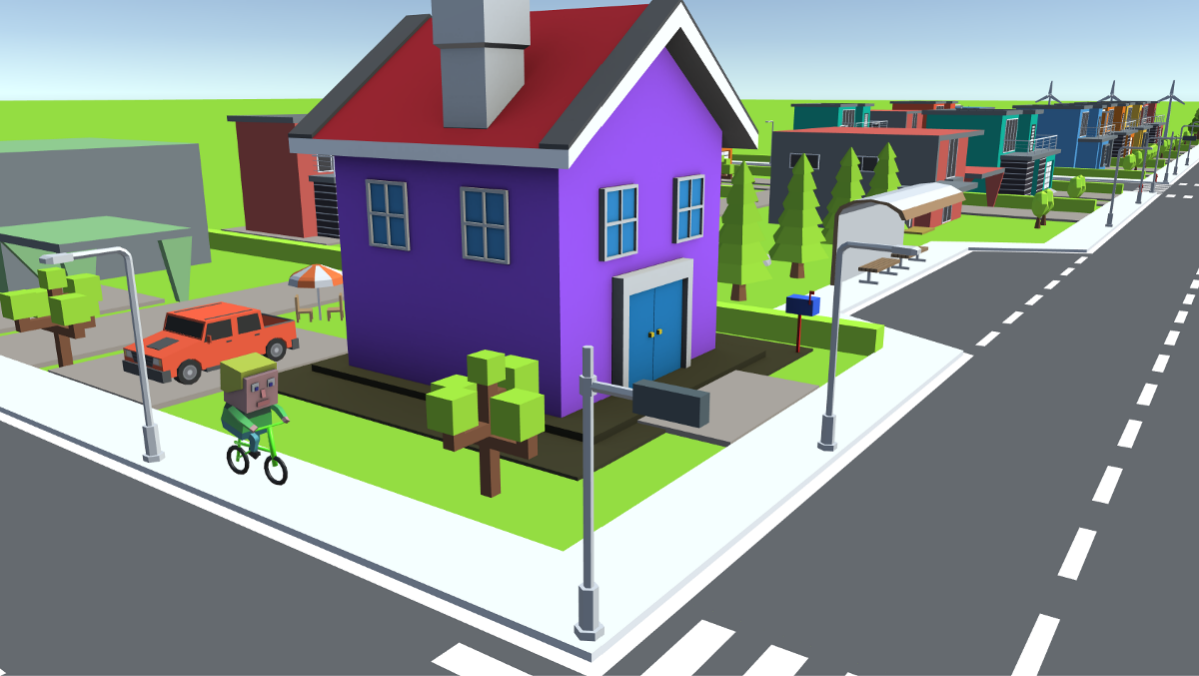
During Gene Adventure, the player carefully manages Eugene’s physical activity to maintain a good lifestyle.

Play-based assessment is implemented through an algorithm that allows us to record changes in the player’s decision-making ability and progress (how they accumulate points and experience and face new issues) during the adventure game session. The system tracks the players’ choices, and the final score is converted into informative and evaluative feedback that is presented to the player at the end of the game session. Based on her or his performance, the player receives positive, intermediate, or negative feedback on 4 aspects: nutrition (Figure 8), physical activity (Figure 9), risk behaviors, and stress management and social interactions.

A composite description of the player’s performance, its implications for health, and a final informative summary of genetic risk management constitute the end of the educational journey. Each game session for a first-time player should last for approximately 30 min.

### Substudy 1

#### Game Evaluation: Serious Game Usability and Engagement

To assess the usability of the game, we defined usability as “the extent to which a product can be used by specified users to achieve specified goals with effectiveness, efficiency and satisfaction in a specified context of use” [24]. According to the literature [25], usability testing is important for the serious game, particularly those designed for heterogeneous populations, including individuals that may not be accustomed to interacting with new technologies (ie, “nongamers”).

Among all the procedures described in literature [26], two main approaches assess usability: an observational analysis, in which a user interacts with the system while the developers observe and note every significant player-game interaction or player’s comment, and a survey-based analysis, in which the user fills out evaluation questionnaires after the game session [27-29]. Although several scales have been developed (eg, the System Usability Scale [30] and the Questionnaire for User Interaction Satisfaction [31]), there are no validated scales to assess games usability thus far. Therefore, we decided to perform a mixed method combining observational analysis of participants and a self-report questionnaire—the Game Experience Questionnaire—to partially overcome the current limitations of both quantitative methods [32].

The observational analysis will record participants’ reactions during the game, with a particular focus on their comments and emotional reaction (laugh, groan, frustration, doubts on how to proceed, etc), to gain real-time information that may not be captured by posttest surveys [33]. The observational analysis will provide information on the overall engagement of the player: Deep engagement in serious games has been associated with learning and students’ academic achievements [34,35].

Engagement refers to a holistic experience or mixture of involvement and enjoyment; it is a key factor for serious games and determines the effectiveness of learning. In previous studies, several measures such as scales of immersion and presence [36], flow [37], and engagement [38] have been applied for different aspects of engagement in serious games. The most-appropriate questionnaire for our serious game is the Game Experience Questionnaire [39] because it measures how players feel during the game session. This questionnaire comprises 33 items and assesses 7 core dimensions: immersion, flow, competence, positive effect, negative effect, tension, and challenge. For each item, participants state their personal experience on a 5-point Likert scale (from 0 indicating not at all to 5 indicating extremely).

#### Target Population and Recruitment

The pilot usability study will be conducted with a convenience sample of 30 participants. All participants will be recruited by e-mail through the students’ institutional mailing list of the University of Milan (Italy; Master Course in Cognitive Science) and via authors’ acquaintances. The e-mail invitation will entail a brief description of the tasks, the medium amount of time required, and the contact information of the experimenter. All the experimental sessions will be conducted in a quiet room at the University of Milan in the presence of an experimenter.

Considering the heterogeneity of potential users, we will enroll 10 participants and categorize them into 2 groups in each game: one group of 5 “young” participants, aged 16-30 years (major users, includes people who play games more frequently), and another group of 5 “middle-aged” participants, aged 31-65 years (minor users of video games). It is important to assess the usability of both age groups since the potential users of our serious game are individuals of all ages.

#### Method and Procedures

Participants will be assigned to one of the three games and will play the game individually. Before interacting with the game, they will complete an informed consent form. Participants will be briefly instructed about the game and prompted to play on their own without any further direction or instruction. In addition, they will be asked to speak loudly during the game in order to communicate their thoughts. At the end of the play session, participants will report any issue, negative or positive, that they encountered during the game and will be invited to complete the Game Experience Questionnaire. The programmers will dedicate time to address and fix the negative issues.

#### Data Analysis

All data on player usability collected from the observational analysis will be examined using text analysis for qualitative data, as reported previously [40]. The negative issues that could compromise the usability of the serious game will be classified on the basis of the usability heuristics developed by Bertini et al [41] (eg, consistency and mapping, ease of input, screen readability and glancability, flexibility, efficiency of use, and personalization) [42]. Nonparametric statistical analysis for quantitative data will be applied to the Game Experience Questionnaire, with group comparisons based on age and technological expertise (gamers vs nongamers; computer and mobile technology users vs nonusers). Statistical analysis will be conducted using the SPSS Software (version 22; IBM Corp, Armonk, NY), with an alpha value of .05 set a priori for all analyses.

### Substudy 2

#### Game Evaluation: Knowledge-Transfer Test

We will assess participants’ learning outcomes to verify the effectiveness of serious games. Most studies evaluated the effectiveness of serious games through the use of pre- and posttest evaluations [43], whereas some studies used a control group of individuals who received the target information through other instructional techniques. We used both assessment methods.

#### Target Population and Recruitment

To test whether the learning goals were achieved, 60 teenagers and adults aged 16-65 years who volunteer to participate will be enrolled: 30 participants will be assigned to the experimental group and 30 participants, to the control group. Both groups will be age- and gender-matched. All participants will be recruited from the general population by using the author’s e-mail and social media contacts, posters emailed to all University of Milan staff and students, and personal invitations and snowball sampling. The invitation will contain a brief description of the tasks, the median time required, and contact information. All the experimental sessions will be conducted in a quiet room at the University of Milan under the supervision of an experimenter.

#### Materials and Procedures

Before starting the game session, participants will complete a demographic questionnaire on age; gender; education level; previous experience with computer, mobile, or tablet devices; and habits of playing video games. Participants with no experience with technological devices will be excluded.

Participants enrolled (experimental and control groups) will complete the following steps: (1) knowledge-transfer pretest questionnaire, (2) self-efficacy pretest questionnaire, (3) serious game-playing session or paper-based information reading, (4) knowledge-transfer posttest, and (5) self-efficacy posttest.

First, an ad-hoc questionnaire will be used to assess genetic topic-specific knowledge, with questions on Mendel’s laws, mutations, and genetic risk implications. The questionnaire comprises multiple-choice questions or true or false questions such as “From a pair of rabbits with Cc and cc genotype respectively, what is the proportion of their children's genotypes: (A) three CC rabbits and one Cc (B) three Cc rabbits and one (CC) two Cc rabbits and two cc (D) four rabbits cc” or “A healthy lifestyle can prevent or lessen the negative consequences of having genetic predispositions to some diseases (True/False).” Participants that correctly answer more than 80% of the questions in the pretest will be excluded from the study, due to the high base-rate literacy in genetics and genetic risk information. Data will be collected using the Lime Survey [44] in a supervised setting in a quiet room at the University of Milan under the supervision of an experimenter.

Second, a questionnaire will be used to assess perceived self-efficacy with regard to knowledge of genetics, defined as confidence in one’s ability to use genetic information. The questionnaire comprises 8 items, 5 of which are taken from the Self-Efficacy Scale in Carrere et al [45] and include items such as “I am able to understand information about how genes can affect my health” and “I am able to explain to others how genes affect one’s health.” In addition, we added one item to assess the perceived knowledge on the interaction of lifestyle and genetic makeup: “I have a good idea about how my own behaviors might interact with my genetic makeup in affecting my health.” These six items are to be answered on a 7-point Likert scale. Further, we added two items to assess the “task-specific” self-efficacy, that is, the self-efficacy participants experienced in answering the knowledge-transfer questionnaire. Self-efficacy is a precursor to the adoption of health-related behaviors [46]. We included its assessment in this protocol, because it is the only measurable parameter to verify the efficacy of serious games in promoting changes in behaviors in our study (we are unable to directly verify participants’ changes in health-related behaviors and decision-making abilities in the light of the new knowledge). Due to the Dunning-Kruger effect [47] (a cognitive bias of illusory superiority), individuals with low literacy in genetics may mistakenly deem their confidence levels to be higher than their actual levels; however, we will be able to match each participant’s expected and real performance, as we will assess their effective knowledge by using the knowledge-transfer questionnaire.

Third, participants will be allocated to the experimental group, where they will play the serious games, or the control group, where they will receive leaflets with the same information given to the experimental group (traditional paper-based approach for learning). Participants in the experimental group will play the two mini-games first (Heredi-Rabbit and Mutan-Tetris) to start from the basic concepts of genetics and proceed with Gene Adventure, which introduces concepts of genetic risk. The overall duration of the game session will be approximately 50 min. The control group will have approximately 50 min to read the genetic information in the leaflets, which is provided by the SCI in the games. Some examples of the content are as follows: “Each gene has at least two variants, called alleles. One allele comes from the mother and the other allele comes from the father” (Figure 1a) or “Some mutations have no effect; some could be auto-fixed by the organism; others could provoke illness, sometime serious (like cancer).” As the primary aim of our study is to investigate the efficacy of the serious games, we believe a media comparison is paramount to determine if knowledge transfer depends on the type of tool used.

Fourth, at the end of the game session, the experimental group and control group will be presented with the posttest questionnaire (with the same questions that were included in the pretest questionnaire) to assess genetic topic-specific knowledge. Significant differences between the test scores in both groups will indicate knowledge-transfer efficacy. Furthermore, the delta of pre- and posttests between the two groups will reveal differences in the efficacy of serious games versus traditional paper-based information.

First, participants from both groups will be asked to fill in the self-efficacy questionnaire again. The differences between pre- and posttest self-efficacy results will reflect the change in confidence of each participant’s knowledge.

#### Data Analysis

For knowledge-transfer analysis, the Cohen *d* effect size will be calculated. Statistical analysis will be conducted using the SPSS Software (version 22; IBM Corp), with an alpha value of .05 set a priori for all analyses.

## Results

In 2017, we collected evidence of game usability. Data have been submitted to the 6th International Conference on Serious Games and Applications for Health by the Institute of Electrical and Electronics Engineers 2018 [42] and used to improve game design. Knowledge-transfer testing will begin in 2018, and we expect to collect data on the learning outcomes of serious games in December 2018.

## Discussion

### Overview

Since the launch of the Human Genome project in 1990, there has been a need to improve genetic literacy among the general public [48]. Overall, genetic literacy refers to the understanding of basic biological mechanisms (eg, knowledge that DNA is an informative molecule and determines our variation and diversity); the personal and health implications of genetics; and the interaction and interdependence of genes, the individual, and the environment [49].

Unfortunately, even well-educated people lack an understanding of these concepts [50,51]. Due to the increasing impact of genetic testing [52] and the importance of decision making in disease prevention, it is crucial to educate people about genetics. Without such knowledge, individuals are more likely to make uninformed decisions or handover all decisions on genetic testing to their doctors.

Technological innovations such as serious games might become valid instruments to support public education and empowerment [50,53-55] and prepare citizens for informed personal and societal decision making in genetics.

Our main endpoint will demonstrate if the use of serious games increases people’s knowledge about genetic mechanisms (eg, prototypes for heredity and mutation) and multiple genetic, behavioral, and environmental factors that contribute to the risk of onset of complex diseases such as heart disease (prototype first scenario in Gene Adventure).

We aim to develop an accessible and simple instrument by representing genetic concepts in an appealing narrative that respects the skills of the general population. With our prototype of Gene Adventure, people can experience, in a simulated life, the management of complex information such as genetic risk and develop a deeper understanding of genetics from experience. If this study proves the efficacy of serious games, the developed serious game could be used in combination with other traditional protocols for genetic counseling to spread awareness for decision making in medical genetics.

### Limits and Future Proposal

This study is designed to test “learning” with reference to information acquisition. The pre- and the posttest questionnaires will measure the performance of correctly answering questions based on new information acquired through the serious game. Further, based on our protocol results, we will test another aspect of learning: the ability to apply newly acquired knowledge in unknown situations by, for example, creating a new scenario in which participants will be asked to make decisions about their own health based on the genetic (risk) issues.

Although there are several other interesting and related themes such as knowledge integration (the ability to integrate information on a given subject derived from different perspectives in a coherent mental representation) [56], this study is not designed to investigate them. At present, our experimental protocol is not sufficiently equipped to differentiate the contribution of each serious game in creating the final mental representation of genetic concepts. However, this study will provide some evidence for this issue through differences between the pre- and posttest performances, which will identify the concepts that participants have understood well, and their achievements during the Gene Adventure game, which will indicate how the users apply their knowledge to increase or decrease their character’s health.

## Abbreviations

DTC: direct to consumer
